# Fenugreek and Okra Polymers as Treatment Agents for the Removal of Microplastics from Water Sources

**DOI:** 10.1021/acsomega.4c07476

**Published:** 2025-04-10

**Authors:** Rajani Srinivasan, Rajita Bhuju, Victoria Chraibi, Mihaela C. Stefan, Nguyen Hien, Damla Ustundag, Jeri La Neice Gill, Nikolas Rasmussen, Blake Saurenmann, Joe Bracerra, Michael Fowler, Hailey White, Marconi Azadah

**Affiliations:** † Department of Chemistry Geosciences and Physics, Tarleton State University, Stephenville, Texas 76402, United States; ‡ Department of Biological Sciences, Tarleton State University, Stephenville, Texas 76402, United States; § Department of Chemistry and Biochemistry, University of Texas at Dallas, Richardson, Texas 75080, United States

## Abstract

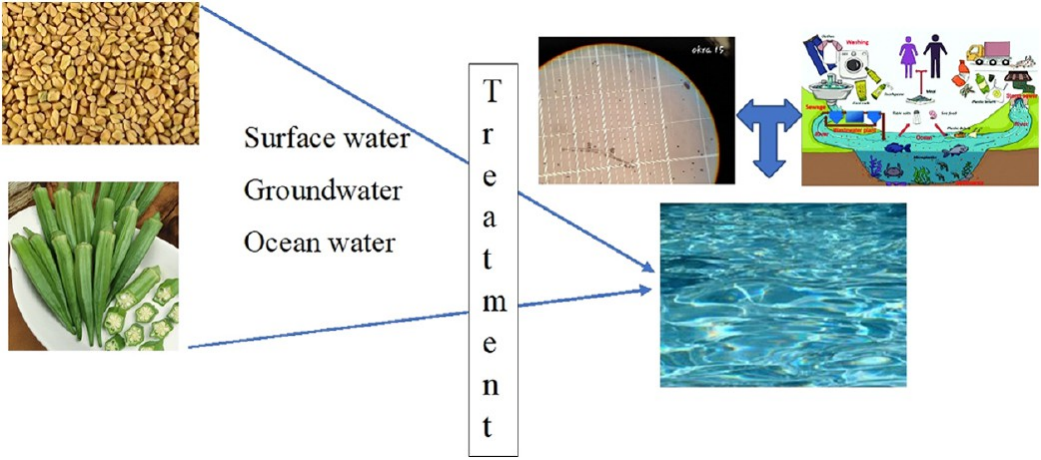

Microplastics originate from the fragmentation of large plastic litter or environmental emissions. These new emerging pollutants not only cause physical harm but also serve as a substrate for other contaminants that adhere to and/or are adsorbed in microplastics. Consumption of these fine particles by organisms may lead to bioaccumulation and bioamplification. Conventional wastewater treatment using inorganic and organic polymeric flocculants is nonbiodegradable and toxic to ecosystem. Plant-derived polysaccharides can provide a highly efficient, nontoxic, and ecofriendly substitute to synthetic flocculants. The microplastic removal efficiency of polysaccharides derived from fenugreek, okra, and the combination of okra and fenugreek in the ratio of 1:1 was studied in simulated and water samples collected from various sources under bench-scale laboratory conditions. Water samples used for the study were collected from surface, ocean, and groundwater sources. A combination of optical microscopy and scanning electron microscopy with energy-dispersive X-ray spectroscopy (EDS) and Fourier transform infrared spectroscopy was used to study the microplastic removal efficiency of the plant-derived polysaccharides. ζ-Potential measurements and scanning electron microscopy were used to confirm the mechanism and capture of microplastic from water samples. The effect of varying polymer concentrations and contact time was also studied. The best concentration was found to be 1 g/L, with fenugreek showing the best microplastic removal in 30–60 min as the optimum contact time. It was found that fenugreek was the most efficient with an ∼89% microplastic removal from groundwater samples. A combination of okra and fenugreek was the most efficient for freshwater samples with an ∼77% microplastic removal. For the ocean water, okra showed the best removal efficiency of ∼80%. The mechanism of microplastic removal using plant-based polysaccharides as flocculant was found to be bridging. Both experimental and statistical analyses demonstrated that plant-based polysaccharides showed better microplastic removal efficiency than polyacrylamide, which is commercially used for water treatment.

## 1 Introduction

Microplastics are a new, emerging contaminant that are becoming detrimental to aquatic environments on a global scale. Microplastics are water-insoluble, solid polymers <5 mm in size. They can be classified into primary and secondary microplastics. Primary microplastics are manufactured directly for personal care use and cosmetic formulations that are used for emulsion stabilization, viscosity regulation, skin conditioning, and synthetic clothes manufacturing.^1^ Primary microplastics are introduced into environments by discharge from industries, water treatment plants, wind deposition, or surface runoff.^2^ Secondary microplastics result from the fragmentation and weathering of larger plastic items by sunlight, wind, and water or by other chemical, biological, or mechanical forces.^3^ Microplastics can threaten the life and development of biota either directly through ingestion or physical irritation or indirectly through the adherence of other pollutants to these particles, which are then ingested and transferred throughout food chains.^4−6^ Thus, effective and inexpensive methods for the detection and removal of microplastics from contaminated water are of great importance. Water and wastewater treatment use a combination of biological and chemical methods to remove pollutants from contaminated waters. Materials that are faster, more efficient, cost-effective, and ecofriendly are highly desirable. Recently, numerous approaches have been studied for the development of cheaper and more effective adsorbents for water treatment. Those containing polysaccharides deserve particular attention. Polysaccharides, including starch, are renewable materials that are widely available and possess biological and chemical properties, including nontoxicity, biocompatibility, biodegradability, and polyfunctionality.

These polysaccharide-based polymers possess chemical and biological properties that aid in the removal of contaminants using the flocculation process. Flocculation is a process that allows the polymers to form a bridge with the contaminants followed by their removal from contaminated water. The polymer particle absorbs the contaminants, becoming heavier in weight, and then settles at the bottom to be later filtered off.^7^ Flocculation usually follows either bridging or charge neutralization mechanism.^8^ Molecular weight (MW) and functional groups are important factors governing flocculation efficiency of the biopolymers. It has been found that biopolymers with higher MW higher than 10^2^ kD follow the bridging mechanism. The presence of specific functional groups like hydroxyl, amide, carboxyl group, amine, etc., enhances the flocculation activity.^9^ Recent studies show that ζ-potential measurements have been used to determine the mechanism of flocculation. Studies have found that flocculation results along with ζ-potential measurements were used to distinguish between the bridging and electrostatic patch mechanisms in water treatment experiments. It was found that reduction in ζ-potential values between the particles increases the flocculation of the particles.^7,10,12^

Coagulation and flocculation by inorganic and organic flocculants modify the physical state of dissolved and suspended solids to form flocs, which sediment out of solution. During the tertiary stage of industrial wastewater treatment, these sedimented solids are removed,^13^ followed by water filtration and disinfection. Potential problems associated with the use of conventional flocculants are the lack of biodegradability and dispersion of monomers or residual polymers in water that may have adverse health effects. Other limitations include their relatively high dosage requirement, high pH sensitivity, and poor efficiency for very fine particle coagulation. Due to their biodegradability, nontoxicity, and easy availability from reproducible agricultural resources, plant-derived flocculants have attracted wide interest from researchers.^13^ Polysaccharides extracted from fenugreek (*Trigonella foenum graecum*),^14,15^ aloe vera (Aloe *barbadensis miller*),^16,17^ okra (*Hibiscus esculentus*),^18^ taro (*Colocasia esculenta*),^19^ and psyllium (*Plantago psyllium*)^20^ have shown promising results as plant-derived flocculants.^16,21^

Plant polysaccharides are composed of long chains of monosaccharide units bound together by glycosidic linkages. Upon hydrolysis, they break down into constituent monosaccharides or oligosaccharides with a variety of functional groups (e.g., carboxyl and hydroxyl groups).^22^ These functional groups serve as binding sites for suspended particles in the adsorption process. Chemically, plant polysaccharides have high chemical reactivity, polyfunctionality, chirality, chelation ability, and high adsorption capacities.^14^ In addition, they can produce flocs, coagulating fine particles, are less sensitive to pH, are effective in both cold and warm waters, and generate less sludge than conventional flocculants.^23^

The contaminant removal efficiency of these polymers is equal to or better than that of existing chitosan-based biomaterials, polyacrylamides, and other synthetic materials.^16^ Bench-scale experiments using these materials in the removal of contaminants from industrial wastewater, surface water, domestic wastewater, and well water by our research group in our laboratory showed lower polymer doses and higher percent removal of contaminants. These materials are capable of removing ∼90–99% of suspended solids and ∼70–75% of total dissolved solids and nutrients. These materials can be used for the purification of wastewater from different sources, with a few changes in their mixing ratios. They can be used as solid polymers or in solution form. The methods used for their isolation from the sources, their formulations, and their use in water treatment are simple, inexpensive, and environmentally benign.^16^

These materials have never been used for water treatment, especially in microplastic removal, before in their present form using the present method. Recent literature shows that scientists are still trying to find better methods for the detection of microplastics^24,7^ and the fate and transport of microplastics in various water bodies, including groundwater.^25^ This research will be a head start in finding ecofriendly materials and methods for removing microplastics from contaminated water using existing infrastructure. This research will be a unique effort in solving the near-future problem of freeing our water from the recent emerging contaminant “**microplastics**”.

## 2 Materials and Methods

### 2.1 Extraction and Characterization of the Polysaccharides

Fenugreek and okra were extracted from its seeds and fruits by overnight soaking in deionized water followed by isopropanol precipitation using the method by Srinivasan and Mishra.^14^ Raw materials, fenugreek seeds, and okra fruits were purchased from the local grocery store. Fenugreek seeds were blended, and okra fruits were chopped into smaller pieces. The chopped pieces and the blended seeds were soaked overnight approximately 3–5 times in deionized (DI) water. The soaked materials were finely blended, the dissolved mucilage was filtered with a muslin cloth, and the remnants were discarded. Then, the mucilage samples were precipitated with 99% isopropyl alcohol in a ratio of one part extracted mucilage solution to three parts of isopropyl alcohol. The precipitated polysaccharides were then obtained using vacuum filtration. The extracted polysaccharides were washed with acetone 2–3 times to remove impurities and dried in a hot air oven at 70 °C (Supporting Figure S1). The extracted polysaccharides were blended into a powder form and stored in a refrigerator at 4 °C for future use.

The prepared polymers were characterized using a Thermo Scientific NICOLET iS10 Fourier transform infrared (FTIR) spectrometer with attenuated total reflection (ATR). A Zeiss EVO LS15 scanning electron microscope (SEM) was used with an Oxford Xplore 30 EDS detector and viscosity method. The polymer samples were dried in an oven at 50 °C and finely ground for FTIR and SEM characterization. FTIR was used to measure infrared spectra of the functional groups of each plant polymer using a spectral range of 4000–500 cm^–1^. The obtained FTIR spectrum was compared with the reference spectral library,^3^ and the background spectrum was recorded. The plant polymers used for the present study are novel, so the FTIR spectra were interpreted based on the properties and structure of the polysaccharides. A small amount of the finely powdered sample was placed on carbon tape attached to the aluminum stub and observed in the SEM. Viscosity was tested using Ostwald’s viscometer by measuring the flow rate and comparing it with the solvent. Each polymer (0.1 g) was dissolved in 100 mL of DI water or 1 molar NaOH to calculate the viscosity average of the molecular weight of the selected polysaccharides.^16^ The okra and fenugreek polysaccharides were sent to the Complex Carbohydrate Research Center located in Georgia Atlanta (CCRC) for molecular weight, composition, and linkage analysis to determine the structural property relationship and mechanism of microplastic flocculation.

Glycosyl composition analysis was performed by combined gas chromatography–mass spectrometry (GC–MS) of the per-*O*-trimethylsilyl (TMS) derivatives of the monosaccharide methyl glycosides produced from the sample by acidic methanolysis and using *myo*-inositol (Inos) as an internal standard. The analysis was performed as described previously by Santander et al.^26^ The MW of the samples was measured using size exclusion chromatography (SEC). For the analysis, 50 mM ammonium acetate was utilized as the running buffer. The sample (1.0 mg) was dissolved in 1 mL of 50 mM ammonium acetate solution by subjecting the resulting mixture to sequential heating and cooling steps as described by McCormick and McCormick et al.^27,28^

### 2.2 Flocculation Experiments

The microplastic removal efficiency of these polymers was tested through bench-scale experiments in simulated and collected water samples. Simulated microplastic-contaminated water was prepared by spiking deionized water (DI) with commercially available microplastic polystyrene beads (10 μm of 2.5% emulsion in water) in the laboratory. Polymer solutions of okra and fenugreek, individually and in combination in a 1:1 ratio with varying concentrations, were prepared. Standard jar tests were used to test the microplastic removal efficiency in both simulated and collected water samples.^16^ The polymer concentrations were varied from 0.5 to 2 g/L. The contact time varied from 5 to 90 min. Water samples were collected from various water sources and locations, such as Houston, Lubbock, and in and around Erath County, TX. Water samples were collected from oceans, wells, and rivers. The collected water samples were brought back to the corresponding author’s (PI) laboratory at Tarleton located in Stephenville TX for experimentation. The collected water samples were characterized for water quality parameters such as pH, total dissolved solids, suspended solids, turbidity, anions, and cations using a pH meter, conductivity meter, turbidity meter, gravimetric analysis, and ion chromatographs in the PI’s laboratory. Qualitative and quantitative microplastic analyses were performed using a Motic Panthera C2 trinocular compound microscope with a 4k digital video camera. Experiments were performed without hydrogen peroxide digestion and repeated with 6–30% hydrogen peroxide digestion^29^ on the collected water samples. It was found that 30% of hydrogen peroxide was able to digest the organic matter in the water samples better. For further experiments, 30% of hydrogen peroxides was added to the water samples in a 1:1 ratio. 10 ml control and treated water samples were collected at different time intervals starting at 0, 15, 30, and 60 min in a 50 mL glass beaker for counting the microplastics. 10 mL of 30% hydrogen peroxide was added to each beaker and placed in an oven at 50 °C for 1 h to digest any competing organic matter. After an hour, the beakers were removed from the oven and cooled. Then, a 10 μL sample was placed in the hemocytometer and microplastics were counted under the microscope under a 20× to 40× magnification (Supporting Figure 2 explaining the method of counting S2). After the completion of the experiments, the treated and untreated water samples were dried in an oven at 50 °C. The solids were scraped and ground into fine particles and observed under the FTIR and SEM to show the adsorption of the microplastics on the polymer surfaces.

### 2.3 Mechanism of Flocculation

Flocculation mechanism was determined using ζ-potential measurement and scanning electron microscopy. ζ-Potential of the polymer solutions and treated and untreated water samples were measured in a Malvern Zetasizer Nano ZS. The jar test was performed using the water samples collected from various water sources. The polymer, contact time and polymer dose were varied. A volume of 50 mL of the supernatant-treated and untreated waters was collected at 5, 15, 30, and 60 min and was used to measure the ζ-potential in the Zetasizer. Finally, the settled flocs were collected and used for ζ-potential measurements. Each measurement was triplicated, and the mean values were reported.

### 2.4 Statistical Analysis

To assess the microplastic removal efficiency of the polymers and to study the effect of the variables like types of polymers, polymer dose, contact time, and source of water samples following statistical analyses were performed. A two-way ANOVA test followed by the post hoc Turkey test were performed using R statistical software for the DI-simulated samples. A two-way ANOVA test and box–whisker plots were used for the water samples collected from surface water, underground water, and ocean water. A *p* value <0.05 was considered statistically significant.

## 3 Results and Discussion

### 3.1 Synthesis and Characterization of the Polysaccharides

Extracted okra (O), fenugreek (F), and fenugreek and okra (FO) polysaccharides were characterized using FTIR. The viscosity average molecular weight was calculated using Ostwald’s viscometer.^16^Table 1 shows the intrinsic viscosity of the used polymers.

**Table 1:** Intrinsic Viscosity of the Used Polymers

name of the polymer	charge on the polymer	intrinsic viscosity (dL/g)
Fenugreek (F)	neutral^15^	10.45
Okra (O)	anionic^13^	4.45
Fenugreek and Okra (FO) (1:1) ratio	unknown	6.45

#### 3.1.1 Glycosyl Composition Analysis of Okra, Fenugreek, and the Combination of Okra and Fenugreek in a 1:1 Ratio

The results of the composition analysis for samples F and O are shown in Table 2. The most abundant monosaccharides present in sample F were mannose and galactose, confirming that sample F contains galactomannan. However, sample O contained galacturonic acid, galactose, and glucose in abundance, suggesting the presence of pectin and glucan. There were less than 5% arabinose, rhamnose, and xylose in both samples. The percentage of carbohydrates in sample F was higher (45.2%) than that in sample O (23.4%).

**Table 2:** Glycosyl Composition Analysis of Sample F and Sample O

	sample F		sample O	
glycosyl residue	mass (μg)	mol %	mass (μg)	mol %
arabinose (Ara)	4.7	2.1	7.0	5.8
rhamnose (Rha)	1.4	0.6	23.2	19.4
xylose (Xyl)	1.8	0.8	3.1	2.6
glucuronic acid (GlcA)			2.2	1.9
galacturonic acid (GalA)	8.1	3.6	25.0	20.6
mannose (Man)	105.2	46.6	1.6	1.4
galactose (Gal)	100.8	44.6	35.0	29.3
glucose (Glc)	3.9	1.7	22.7	19.0
total sugar	225.8	100	119.2	100
sample mass (μg)	500.0		500.0	
carbohydrate content (%)	**40.7**		**21.5**	

#### 3.1.2 Uronic Acid Linkage Analysis

**Figure 1:**
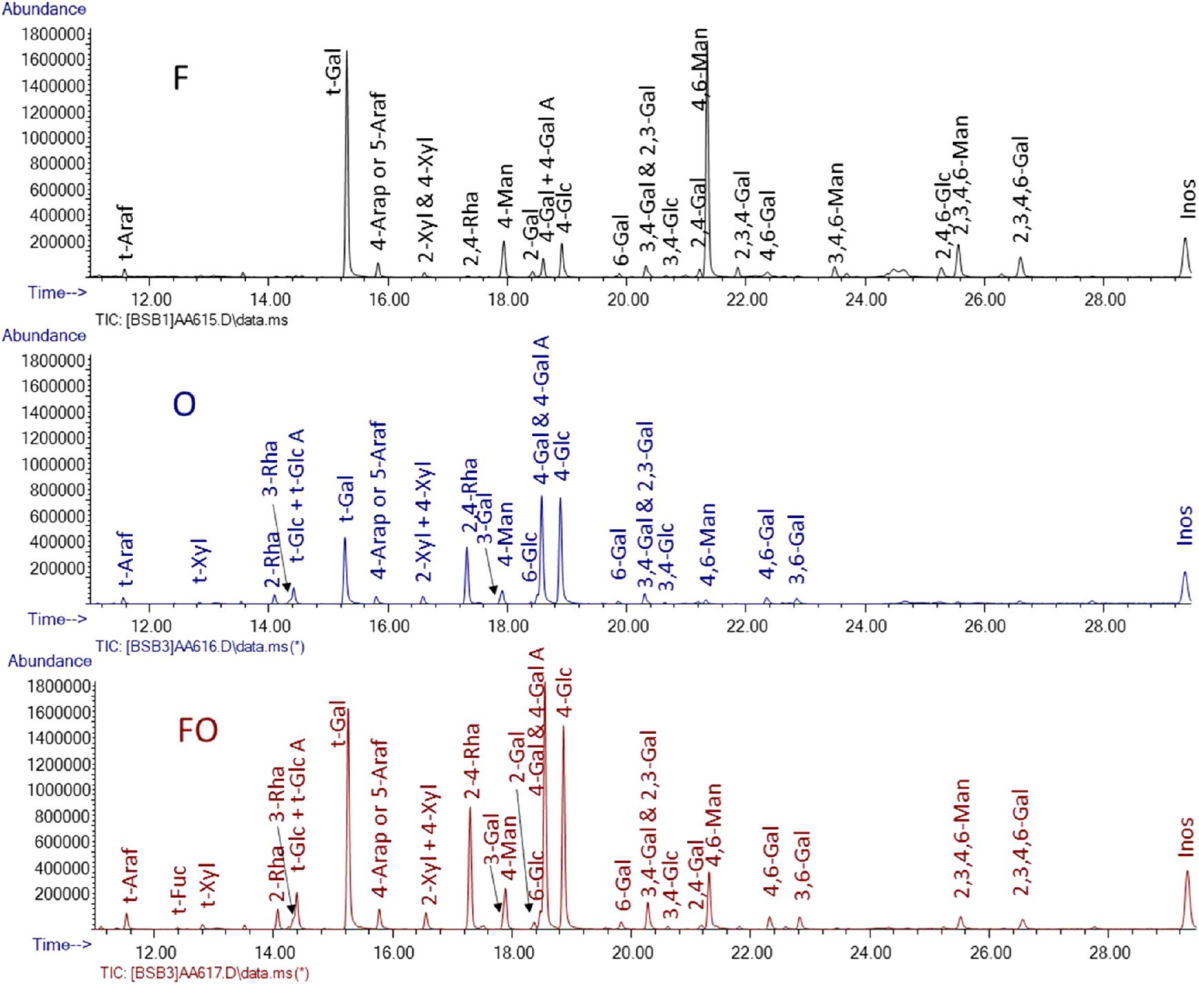
GC chromatogram of linkages detected in samples F, O, and FO.

The results of the linkage analysis of three samples (F, O, FO) are shown in Figure 1. During permethylation, the samples were not fully permethylated after three rounds of NaOH base and iodomethane addition, as seen during DCM extraction, and there were some insoluble samples at the DCM and H_2_O interfaces. Therefore, one more round of permethylation was carried out to facilitate the complete permethylation of the samples. The most abundant PMAAs corresponded to terminal galactose in samples F and FO. However, sample O had 4-linked glucose in high abundance. The fact that the percentage of 4-Glc was not much higher than the glucose percentage in the composition analysis indicates that the glucose came from starch rather than cellulose. Samples O and FO had significantly higher levels of 2,4-linked rhamnose than F. These results and the detection of 4-GalA are consistent with the composition analysis and suggest the presence of rhamnogalacturonan I.

### 3.2 Batch Experiments with Simulated Water Samples

The results described are the means of six experimental replications. The results showed the effect of F, O, and FO polymers at 1 g/L of concentration on the removal of microplastics from water samples.

**Figure 2:**
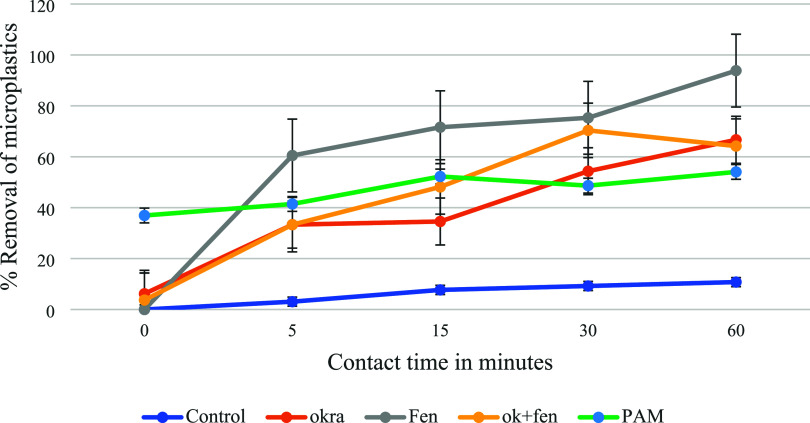
Plots showing the % removal of polystyrene in simulated water samples at varying contact times using fenugreek (Fen), okra (ok), a combination of fenugreek and okra in a 1:1 ratio (ok + fen), and polyacrylamide (PAM).

Figure 2 shows the results of the batch experiments. It was found that 1 g/L of the polymer solution showed maximum removal efficiency, and the optimum time was found to be 60 min. A total of 66.67% removal was shown by okra in 60 min of contact time. Fenugreek showed a 93% removal in 60 min, and fenugreek and okra in a 1:1 ratio showed a 70% removal in 30 min. Comparatively, polyacrylamide removal showed a 54% removal at 60 min. From the viscosity measurements, it was found that F has the maximum viscosity followed by a combination of FO and then O. This might be the contributing factor for better microplastic efficiency of F as compared to other polymers. Higher intrinsic viscosity and higher molecular weight aid in better entanglement of flocculant with microplastics, thus increasing the removal efficiency.^30^

### 3.3 Fourier Transform Infrared Spectroscopy (FTIR) for Simulated Water Samples

The interaction of the polymers with the microplastic polystyrene was confirmed by FTIR and compared with the standard library. The FTIR spectra showed the interaction of the polymers with polystyrene in the treated simulated water sample. Example FTIR spectra for O, microplastics, and simulated wastewater samples treated with the O show the interaction of the polymers with the microplastics.

The FTIR spectra of the O polymer-treated simulated wastewater sample show the two distinct peaks of polystyrene at 2843 cm^– 1^ of the −CH stretch and at 1345 cm^–1^ of the −CH bend from the FTIR spectra of polystyrene. It also shows the presence of peaks at 3680 cm^–1^ of the −OH stretch, at 2980, 2922, and 2865 cm^–1^ of the −CH stretch and at 1454 cm^–1^ of the −CH bend, which were common peaks in the FTIR spectra of O polymer. This shows the interaction between the O and microplastics at 60 min in the treated water sample (Supporting Figure S3).

### 3.4 Batch Experiments with Water Samples Collected from the Ocean, River, and Groundwater

**Figure 3:**
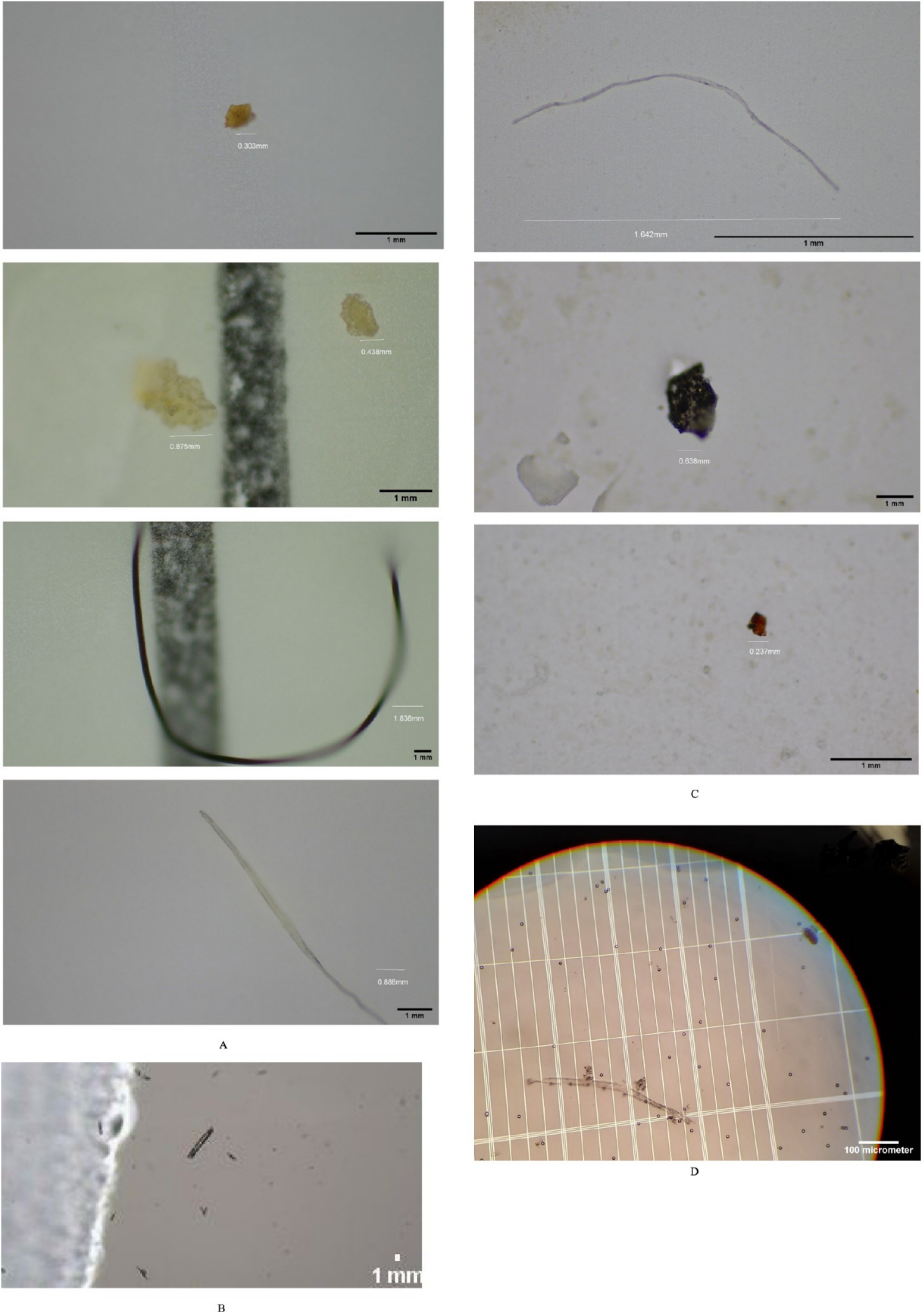
(A) Types of microplastics in Colorado River under 20× magnification. (B) Types of microplastic in well water samples under 40× magnification (C) Types of microplastic in Port Lavaca samples under 20× magnification. (D) Floc formation between the polymer and polystyrene at 30 min of contact time at 10× magnification when treated with okra polymer.

Water samples were collected from Colorado River located in the Timberlake biological field station in Goldthwaite, TX, Port Lavaca in Matagorda Bay near Houston, TX, and well water from Lubbock, TX. A jar test was performed on water samples collected from various locations. Individual polymers and their combinations at various concentrations were used. Concentrations were varied from 0.5 mg/L to 2 g/L. Contact times were varied from 5 to 60 min. The water samples had different types of microplastics with different colors, shapes, and sizes. Microplastics ranged from small particles to long fibers (Figure 3). Microplastics were manually counted under the microscope at different magnifications using a hemocytometer. Manual counting of the microplastics is tedious but was found to be more accurate due to the various shapes and sizes of the microplastics in the water samples (Sun et al.).^3^ To reduce the error for duplicate counting, the microplastic solution was put in a hemocytometer. The Nile red dye method^31^ did not work for our study since the dye also labeled the polymers. Table 3 shows the maximum removal of microplastics from various water samples using single and combinations of various plant-based polymers described above. It was found that 1 g/L and 60 min were the optimum polymer concentration and contact time. The table also provides the comparative data for polyacrylamide at the same polymer concentration and contact time. From Table 3, it was found that the best polymer individually and in combination changes with the type of water used. It was found that F individually showed better removal efficiency from groundwater, ranging from 80 to 90%. FO was the most efficient for freshwater samples, with an approximately 77% microplastic removal. For the ocean water from Port Lavaca, O showed the best removal efficiency of ∼80%. This may be because of the type of different microplastics present in each water sample and the affinity of the various groups present in the polymer samples irrespective of the viscosity of the polymer.^30^ In the most recent experiments, the jar test was repeated with water samples in which 30% hydrogen peroxide was used to digest competing organic materials in the water samples treated with F,O and FO before microplastic counting. Similar results were obtained. It was much easier to count the microplastics as compared to samples without hydrogen peroxide digestion.

**Table 3:** Comparison of % Removal of Microplastic, Polysaccharide Polymer (PP) vs Polyacrylamide (PAM) in Water Samples

name of the polymer	water sample	contact time (min)	maximum% removal of microplastic	amount used	corresponding PAM microplastic removal
Fenugreek	Well 17	60	89	1 g	43
Okra	Well 17	60	85.7	1 g	61.5
Okra: Fenugreek	Well 17	60	75	1 g	43.7
Okra: Fenugreek	Well 15	15	66.67	1 g	64.86
Fenugreek	Well 16	60	46.15	1 g/L	53.85
Fenugreek	Colorado River	60	47.8	1 g/L	33.33 (30 min)
Okra	Colorado River	60	68.18	1 g/L	47.05
Okra	Colorado River	60	61.48	1 g/L	61.63
Fenugreek	Colorado River	60	30.07	1 g/L	44.09
Fenugreek: Okra	Colorado River	60	77.27	1 g/L	40.9
Fenugreek	Port Lavaca	15	52.54	1 g/L	41
Okra	Port Lavaca	60	81.81	1 g/L	45.45
Okra	Port Lavaca	30	45.54	0.5 g/L	50
Okra: Fenugreek	Port Lavaca	30	58	1 g/L	25

### 3.5 Fourier Transform Infrared Spectroscopy (FTIR) of Water Samples

**Figure 4:**
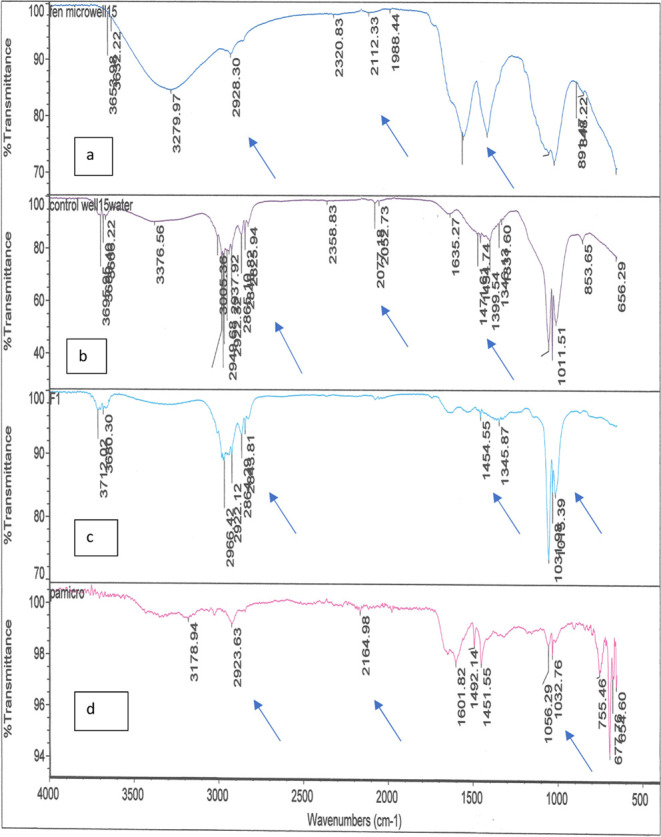
Comparison of the FTIR: (a) well water treated with fenugreek, (b) control well water, (c) fenugreek polymer, and (d) well water treated by polyacrylamide.

Figure 4a–d shows the FTIR analysis of well water samples with and without treatment with F polymer in comparison to polyacrylamide polymer. Figure 4a represents FTIR of the treated well water with F, followed by Figure 4b, control well water without treatment; Figure 4c shows the FTIR of the F, and Figure 4d shows the FTIR of the well water treated with polyacrylamide. Comparing Figure 4a–c, the arrows indicate the adsorption of the microplastics from the water by the F. Comparing Figure 4a,d, it can be inferred that F was capable of adsorbing more microplastics as compared to polyacrylamide, which is indicated in Table 3. Figure 4b shows the FTIR spectrum of the well water. The spectrum shows similar peaks when compared to pure FTIR spectrum of polyvinyl chloride (PVC) from the literature, showing the presence of PVC in the well water sample. A slight shift in the peaks may be due to the interaction with other materials in the water samples. A pure PVC spectrum obtained from the literature shows peaks at 297 and 2910 cm ^–1^ consisting of the CH_2_ asymmetric stretching vibration mode. The peak at a higher wavenumber shows the asymmetric stretching bond of C–H, and the lower peak is for the symmetrical stretching bond of C–H. The peaks around 1400 cm ^–1^ are assigned to the C–H aliphatic bending bond. The peak at 1250 cm ^–1^ is attributed to the bending bond of C–H near Cl. The C–C stretching bond of the PVC backbone chain occurs in the range of 1000–1100 cm ^–1^. Finally, peaks in the range of 600–650 cm^–1^ correspond to the C–Cl gauche bond.^32^Figure 4c represents the F, which is a galactomannan. The FTIR spectrum shows peaks of –OH between 3609 and 3288 cm^–1^, ether linkage at 1455–1400 cm^–1^, −CH stretching between 2923 and 2854 cm^–1^, −C–O stretching at 1018 cm^–1^, and −CH_3_ stretching at 2923 cm^–1^.^14^Figure 4a,d shows the capture of PVC by the F and polyacrylamide polymer. It can be seen that the prominent peaks of PVC can be found in Figure 4a,d. Figure 4d shows an intense peak around 1600 cm^–1^, depicting the presence of –C=O and NH_2_ peak around 3178.94, indicating the presence of polyacrylamide polymer.

### 3.6 Statistical Analysis

#### 3.6.1 Simulated Water

##### 3.6.1.1 Determination of the Optimal Polymer Dose for the Removal of Microplastics from Water Samples

Two-way ANOVA showed significant differences among the means of various concentrations of polysaccharides. Similarly, a post hoc Tukey test was performed to determine differences among the groups and revealed significant differences between the various concentrations of polymers and controls. However, there were no significant differences among the three concentrations of polymer.

##### 3.6.1.2 Determination of the Efficacy of Different Polymers in the Removal of Microplastics from Water Samples

The results of two-way ANOVA showed significant differences among the means of the three polymers. The results from the post hoc Tukey test showed significant differences between the means of all three polymers and controls. These results indicated that these polymers (F, O, and FO) were efficient in the removal of microplastics from water samples. However, for differences in means among the three polymers, no significant difference was found. Overall, statistical analysis showed promising results, demonstrating that all three plant polymers were efficient in the removal of microplastics from the water sample and was dependent on the sedimentation time.

Tables 4 and 5 show the ANOVA and post hoc Turkey test for okra with polystyrene in simulated water.

**Table 4:** Two-Way ANOVA Test between the Mean of Different Concentrations of Okra Polymer

	Df	sum of squares	mean square	*F* value	Pr (>F)	significance level
polymer	3	3767	1255.7	15.19	0.000362	0.05
residuals	15	1240	82.7			

**Table 5:** Post Hoc Tukey Test for the Analysis of Difference Occurred between Different Concentrations of Okra Polymers

variable	estimate	SE	df	*t* ratio	*P* value
0.01 g/L—control	–31.2	5.25	15	–5.937	0.0001
0.02 g/L—control	–29.2	5.25	15	–5.556	0.0003
0.04 g/L—control	–25.2	5.25	15	–4.794	0.0012
0.01–0.02 g/L	–2.0	5.25	15	1.415	0.5096
0.02–0.04 g/L	–4	5.25	15	–0.762	0.8702
0.01–0.04 g/L	–4.17	5.25	15	1.415	0.5096

#### 3.6.2 Collected Water Samples

Statistical analysis was performed using confidence intervals with a 95% level of significance and box–whisker plots. Two difference analyses were performed to compare the efficiency of the polymers with that of polyacrylamide. The microplastic removal efficiency was compared between the water samples and between the polymers. In all cases, polysaccharide-based polymers performed better than polyacrylamide. Two-factor ANOVA between the polymers showed no significant difference between and within the polymers in the microplastic removal efficiency in water samples. However, there was a significant difference in the microplastic removal efficiency between the polymers and polyacrylamide, with a *P* value of 0.01 and an *F* value of 8.07 compared to *F* critical = 4.74. A single-factor ANOVA between the Colorado River and well water samples showed a significant difference in the microplastic removal efficiency, as indicated by the *F* value: 7.63, P value: 0.03, and *F* critical value: 5.98. It was found that polymers performed better in microplastic removal in well water than in freshwater samples collected from the Colorado River. This may be because of the type of microplastics present in the two different water samples.

##### 3.6.2.1 Analysis between the Water Samples

##### 3.6.2.1.1 Fresh Water from Colorado River

**Figure 5:**
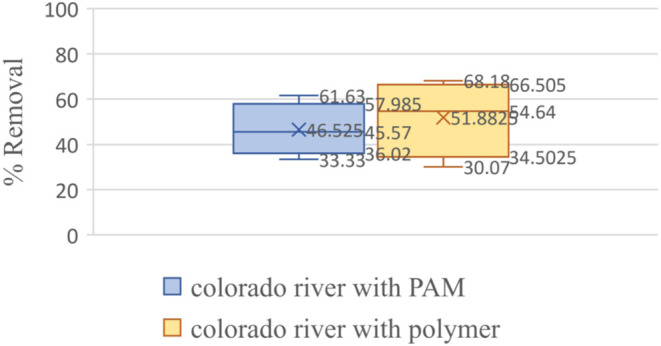
Box–whisker plot showing the statistical significance of the microplastic removal efficiency plant-based polymers as compared to that of polyacrylamide in Colorado River.

The polysaccharide-based polymers showed a range of 36–68%, and polyacrylamide showed a 35–58% microplastic removal from water samples collected from the Colorado River (Figure 5).

##### 3.6.2.1.2 Ocean Water from Port Lavaca

**Figure 6:**
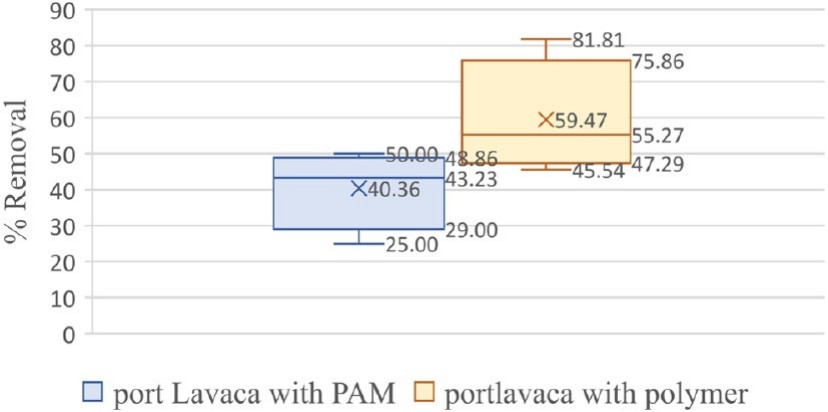
Box–whisker plot showing the statistical significance of the microplastic removal efficiency plant-based polymers as compared to polyacrylamide in Port Lavaca.

The polysaccharide-based polymers showed a range of 44–75%, and polyacrylamide showed a 30–51% microplastic removal from water samples collected from Port Lavaca near Houston, TX (Figure 6).

##### 3.6.2.1.3 Underground Water Sample from Lubbock

**Figure 7:**
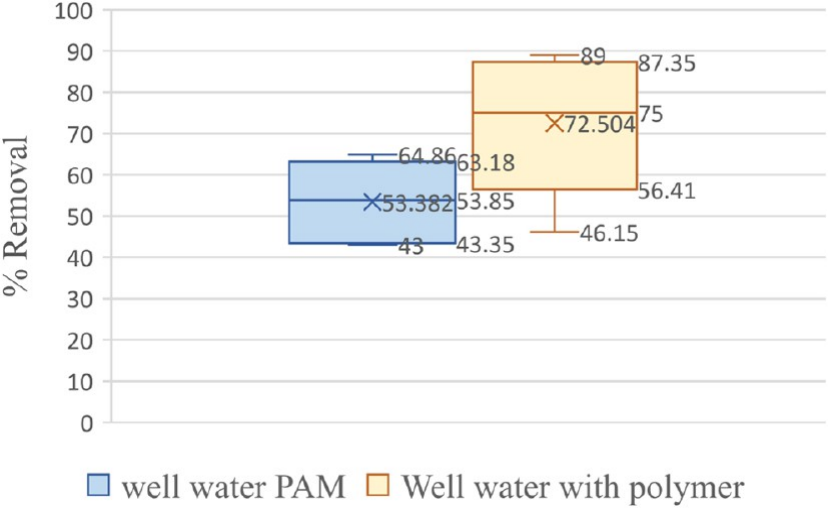
Box–whisker plot showing the statistical significance of the microplastic removal efficiency plant-based polymers as compared to polyacrylamide in well water samples.

The polysaccharide-based polymers showed a range of 57–88%, and polyacrylamide showed a 45–62% microplastic removal from water samples collected from well water from Lubbock, TX (Figure 7).

##### 3.6.2.2 Analysis between the Polymer Samples in Comparison to Polyacrylamide

**Figure 8:**
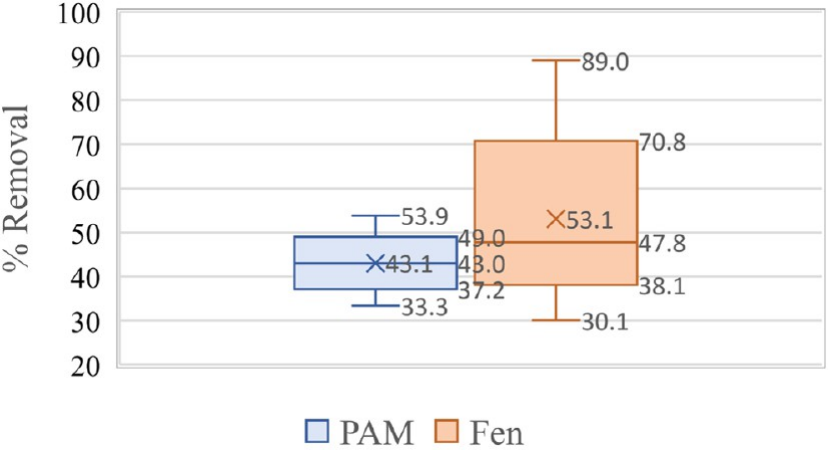
Box–whisker plot showing the statistical significance of the microplastic removal efficiency of fenugreek and polyacrylamide in various water samples.

**Figure 9:**
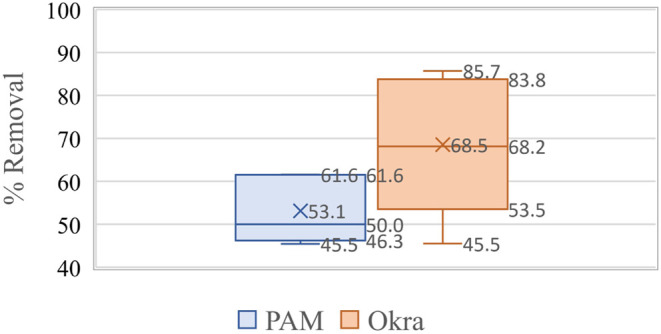
Box–whisker plot showing the statistical significance of the microplastic removal efficiency of okra and polyacrylamide in various water samples.

**Figure 10:**
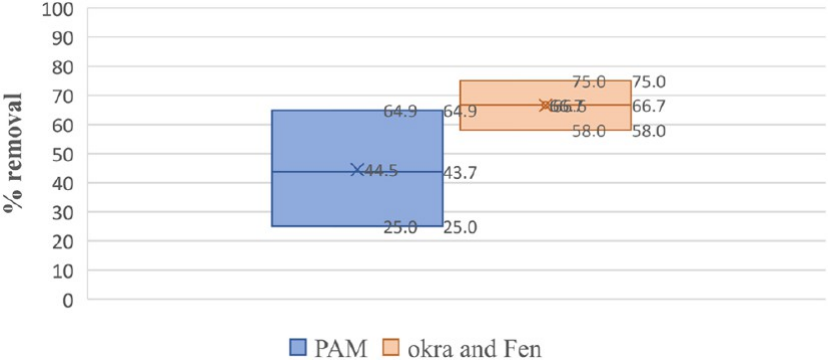
Box–whisker plot showing the statistical significance of the microplastic removal efficiency of a combination of fenugreek and okra and polyacrylamide in various water samples.

Polymers show a better removal efficiency than polyacrylamide with 95% significance as per statistical analysis. O showed a lower to upper confidence limit of 54–82% and polyacrylamide showed a 46–60% microplastic removal in all three types of water samples used in the study. F showed lower and upper confidence limits of 32–74% compared to polyacrylamide, which showed a 37–50% microplastic removal. FO showed a lower to upper confidence interval of 57–76% compared to polyacrylamide, which showed a lower to upper confidence interval of 22–67% microplastic removal in all three water samples. Figures 8, 9 and 10 show the same trend.

## 4 Mechanism of Flocculation

**Figure 11:**
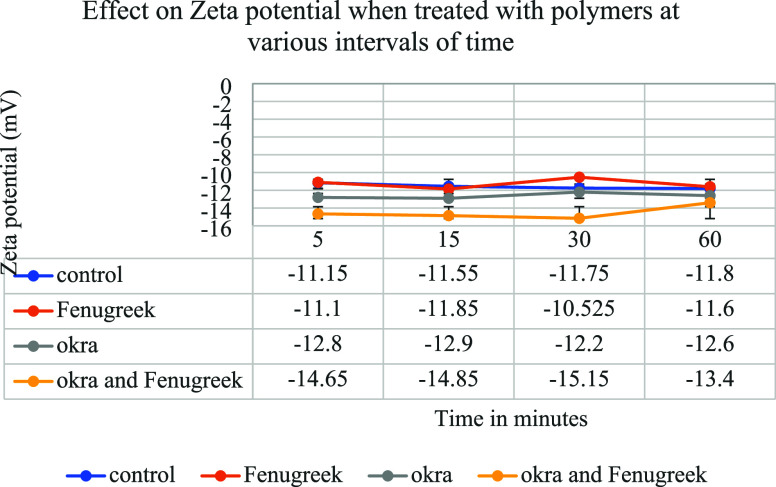
Plot showing the effect on ζ-potential values when treated with various polymers compared to control.

The proposed mechanism for the flocculation of microplastics by the polysaccharides is bridging. Figure 3D shows the bridging process when observed under the microscope in our laboratory. As per the literature, polysaccharides with molecular weights greater than 10^2^ kD along with suitable functional groups confirmed by FTIR show a bridging mechanism for flocculation.^33^ As mentioned above, the molecular weights of the F and O used in the study were found to be greater than 500 kDa, which is greater than 10^2^ kD, proving the proposed bridging mechanisms. The flocculation mechanism based on the ζ-potential measurements also shows that microplastic removal using plant-based materials follows the bridging mechanism. Figure 11 shows the ζ-potential values conducted on the Colorado River water samples before and after treatment. Figure 11 shows that the ζ-potential values between the particles are very low, which help in improving the flocculation efficiency when treated with the polymers. From the figure, it was also found that there was a very slight variation in the ζ-potential values when comparing the water samples before and after treatment with the polymers.^10,11,34^ This also supports the bridging mechanism. If there is a huge change in the ζ-potential value after adding the flocculants, it would support the charge patch mechanism. Based on the optical microscopy and ζ-potential measurement, the removal of microplastics using polysaccharide-based polymers follows the bridging mechanism.

### 4.1 Scanning Electron Microscopy (SEM)

**Figure 12:**
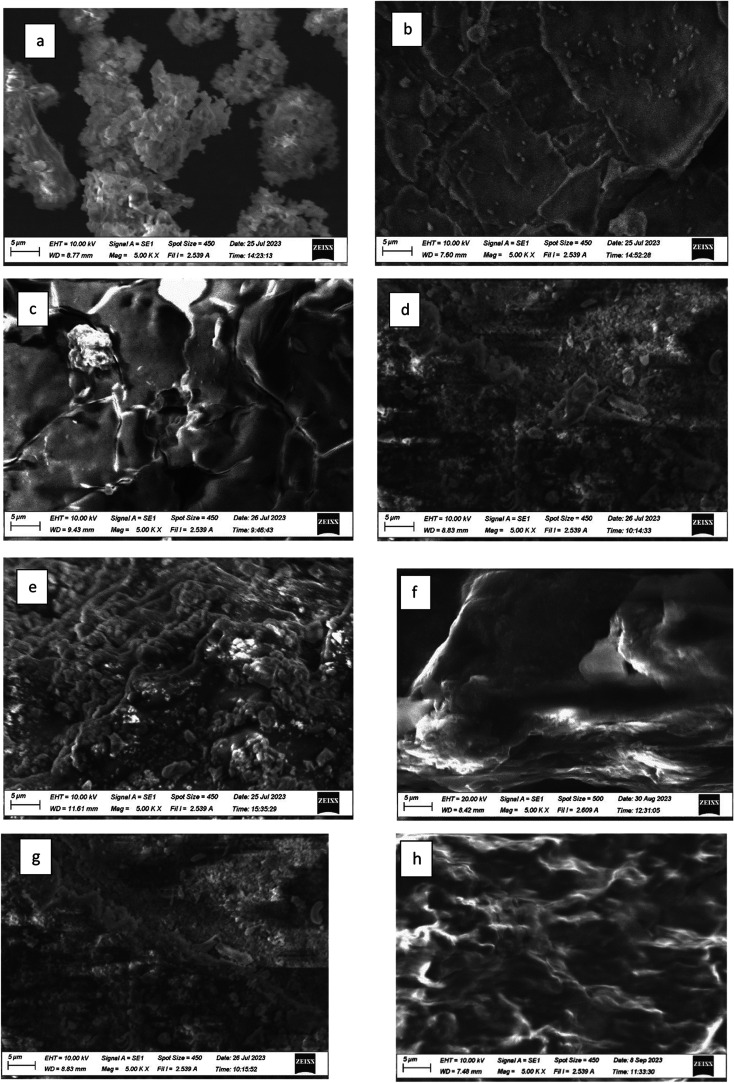
SEM pictures showing the absorption of contaminants from (a) well water, (b) combination of fenugreek and okra adsorbing the microplastics, (c) fenugreek adsorbing the microplastics, and (d) okra adsorbing the microplastic. (e) Polyacrylamide adsorbing the microplastics, (f) fenugreek, (g) okra, and (h) fenugreek and okra.

**Figure 13:**
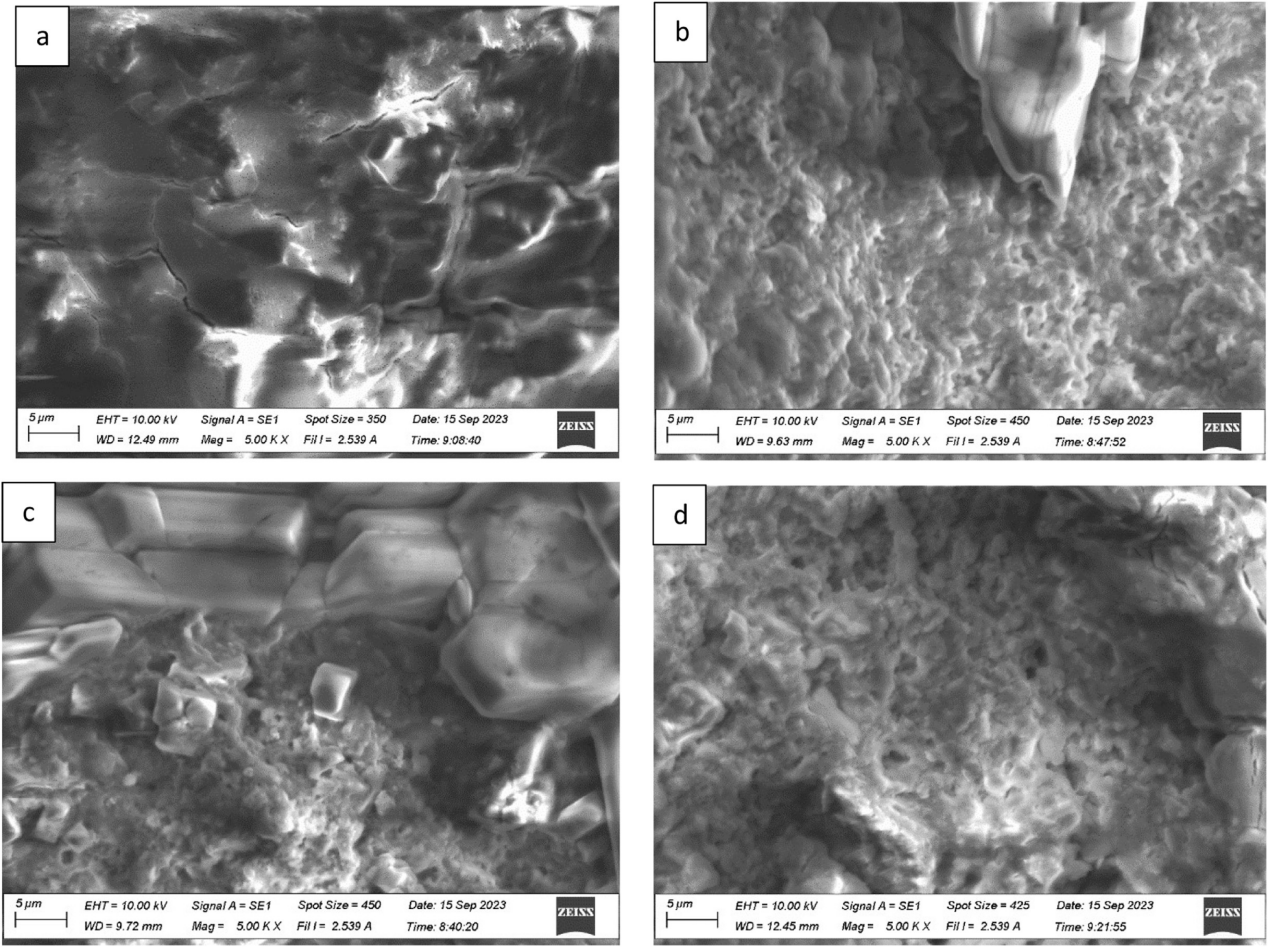
SEM pictures showing the absorption of contaminants from Port Lavaca (H_2_O_2_): (a) Port Lavaca, (b) fenugreek and okra in a 1:1 ratio, (c) fenugreek, and (d) okra.

**Figure 14:**
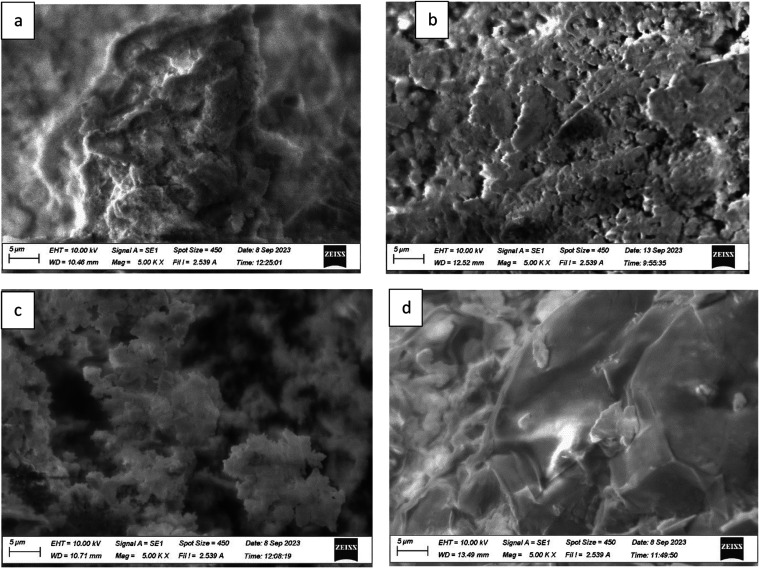
SEM pictures showing the absorption of contaminants from Colorado River, TX: (a) Colorado River, (b) combination of fenugreek and okra (1:1), (c) fenugreek, (d) okra, and (e) polyacrylamide.

**Figure 15:**
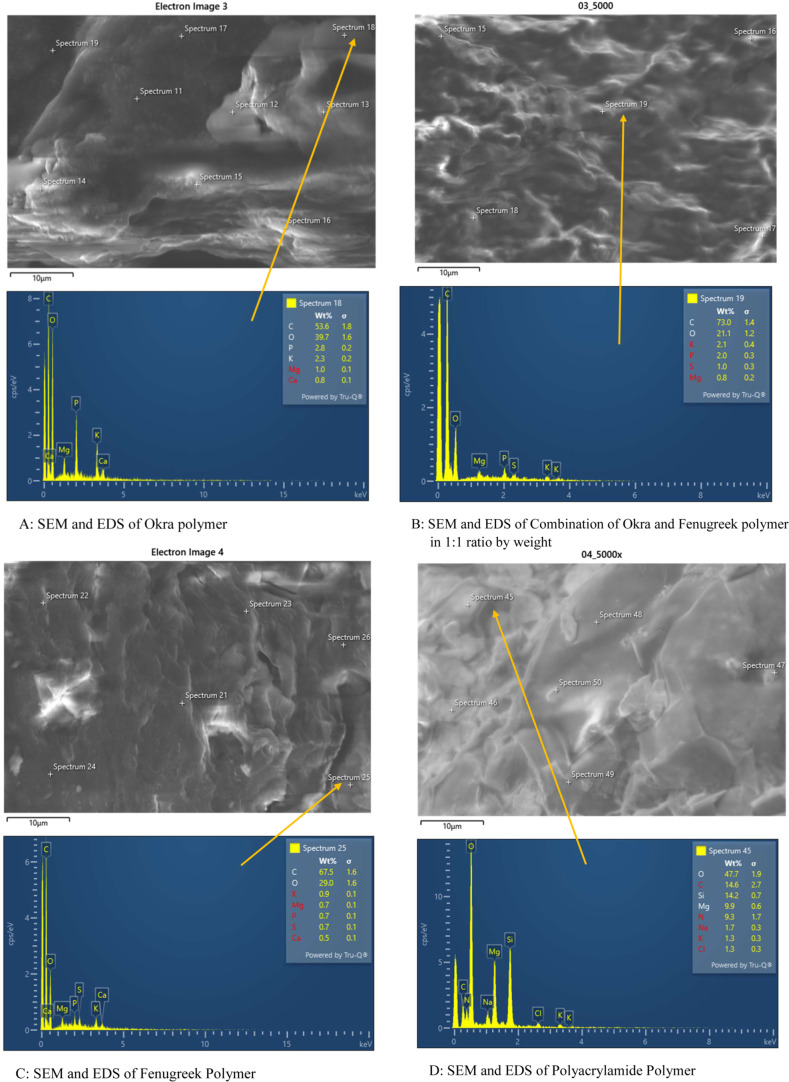
SEM figures with EDS showing the polymers used for the treatment in the study.

**Figure 16:**
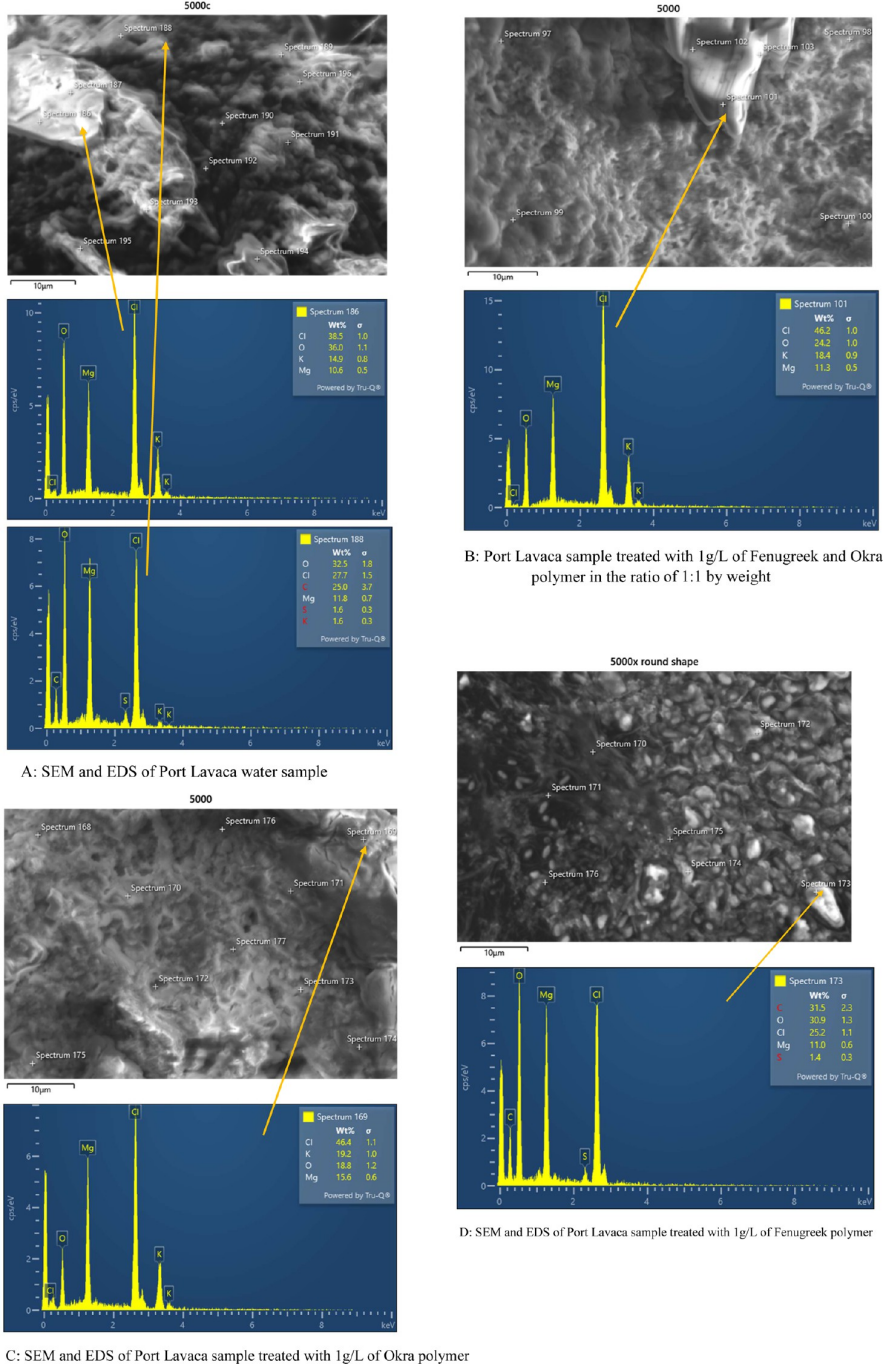
Comparison of the SEM figures with EDS showing the capture of microplastics in Port Lavaca water samples.

**Figure 17:**
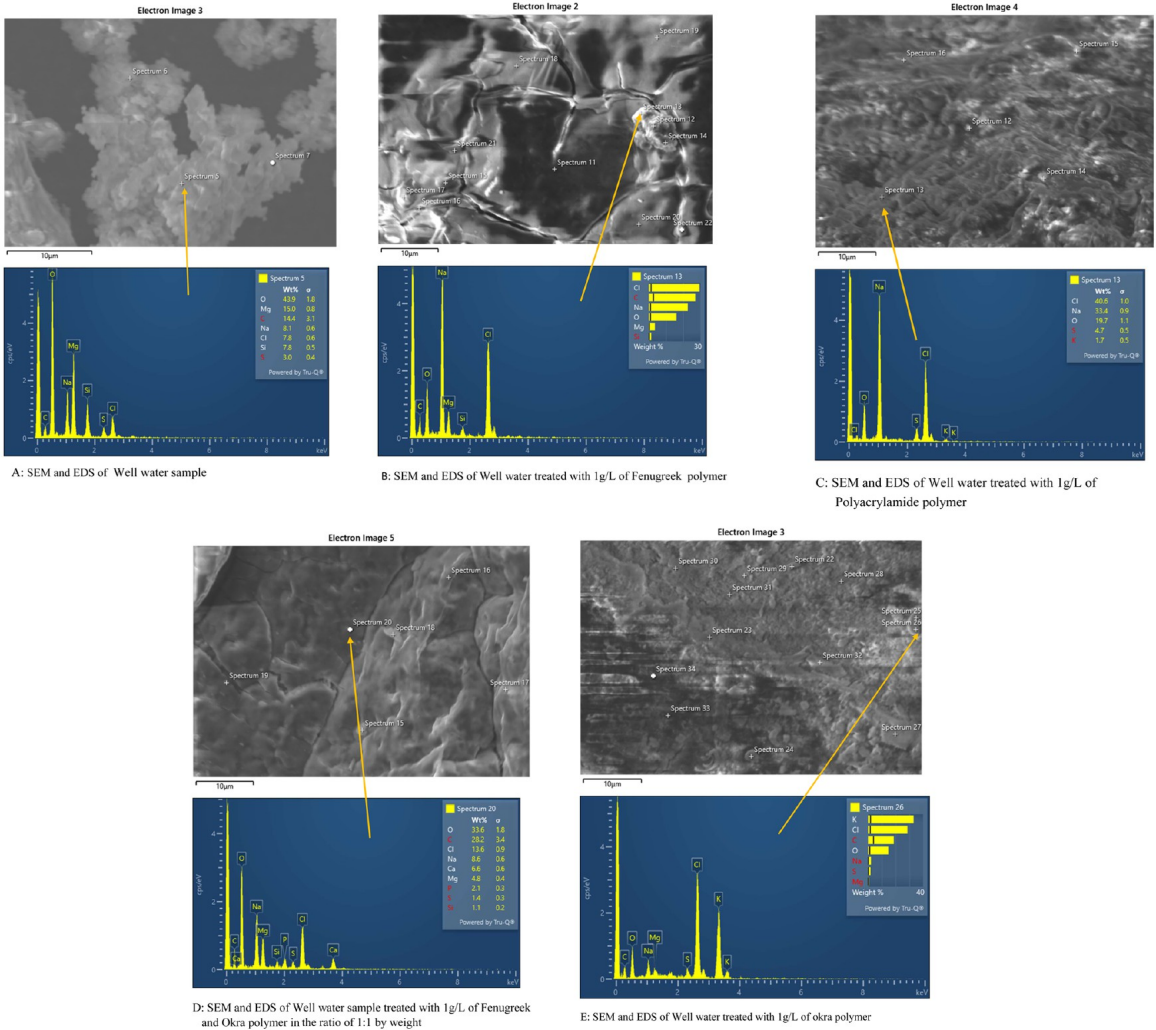
Comparison of the SEM figures with EDS showing the capture of microplastics in well water samples.

**Figure 18:**
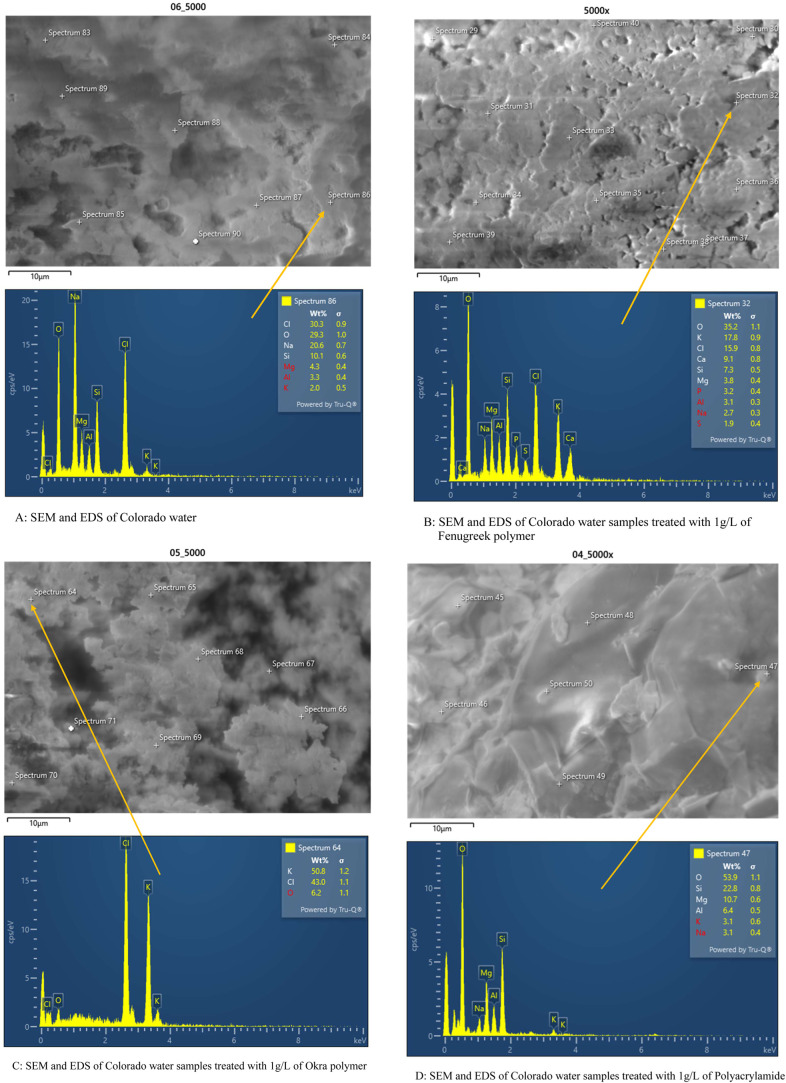
Comparison of the SEM figures with EDS showing the capture of microplastics in Colorado River samples.

SEM was used to show the capture of microplastics by the F, O, FO, and polyacrylamide polymers. EDS data was used to identify some of the types of microplastics present in the water samples. The work is ongoing to determine the specific microplastics present in the water samples. Figures 12, 13, and 14 show the SEM images of treated and untreated water samples along with the SEM pictures of F, O, FO, and polyacrylamide polymers. The SEM pictures show the images at a scale of 5 μm. Figures 15, 16, 17, and 18 show the EDS data corresponding to the SEM images. Figures 15–18 show the SEM figures along with the EDS data of polymers used, Port Lavaca water samples before and after treatment, well water samples before and after the treatment, and Colorado water samples before and after the treatment, respectively. The yellow arrow indicates the part of the spectra in the SEM images for which EDS data is included. The EDS data can be used to identify specific types of microplastics that have been adsorbed by the polymers. Some plastics like polyvinyl chloride (PVC), polyphenylene ether (PPE), polystyrene, etc., can be identified using this method by Wang et al.^35^ Comparing the SEM figures with the EDS data in Figures 12–18, it was found that the polymers used in the study were able to capture one of the common microplastics like polyvinyl chloride, which was identified with the presence of high chlorine peak in the EDS, which was absent in the polymer samples but was present in the untreated water samples and flocs of the treated samples. Comparison with the FTIR spectrum confirms the presence of PVC in the water samples as indicated by the EDS values in SEM images. Optical microscopy helps in segregating microplastics from the samples. Combining SEM with EDS helps in further distinguishing and confirming the morphology, structure, and behavior of both polymers, microplastics, and their capture by the polymer, thus reducing any misidentification.^35^

Based on the study, Table 6 summarizes the efficiency of the polymers used in different types of water samples used in the study.

**Table 6:** Summary of the Maximum % Removal of Microplastics in Different Types of Water Samples Used in the Study

type of water samples	maximum % removal of microplastics	type of polysaccharide polymer used
simulated water samples	∼93	Fenugreek
surface water samples	∼77	combination of okra and fenugreek in a 1:1 ratio
underground water samples	∼89	Fenugreek
ocean water samples	∼80	Okra

## 5 Conclusions

From the experimental and statistical analyses, it is concluded that polysaccharide-based polymers showed a better microplastic removal efficiency than the commercially available polyacrylamide. The best concentration was found to be 1 g/L, with fenugreek showing the best microplastic removal. FTIR and microscopic analyses show the interaction of the polymers with the microplastics. The most appropriate mechanism was found to be bridging based on molecular weight determination, microscopic pictures, and ζ-potential values. Maximum polystyrene removal by fenugreek in simulated water samples is due to its high intrinsic viscosity of fenugreek polymer. It was found that F individually showed better removal efficiency from groundwater, ranging from 80 to 90%. FO was the most efficient for freshwater samples, with an ∼77% microplastic removal. For the ocean water from Port Lavaca, O showed the best removal efficiency of ∼ 80%. This may be because of the different types of microplastics present in the water sources and their affinity toward the various groups in the polymers. More experiments are being performed to determine the type of microplastics in different types of water sources and their interaction with the polymers.

## Supporting Information Available

Plant-derived polymer extraction method (Figure S1); microplastic counting method using a hemocytometer (Figure S2); and FTIR spectra of simulated water with polystyrene treated and untreated with okra polymer (Figure S3) (PDF)
