# Supplementary material for: Fenugreek and Okra Polymers as Treatment Agents for the Removal of Microplastics from Water Sources
Source: ACS Omega. 2025 Apr 10;10(15):14640–56. doi: 10.1021/acsomega.4c07476 (PMC12019522; doi:10.1021/acsomega.4c07476)
Supplement: Supplementary file 1 — ao4c07476_si_001.pdf [file ao4c07476_si_001.pdf]

Supplemental Information (For Review only)

Title Page

Fenugreek and okra polymers as treatment agents for the removal of microplastics from water sources.

Rajani Srinivasan[*a], Rajita Bhuju[a], Victoria Chraibi[b], Mihaela C. Stefan[c], NguyenHien [c], Damla Ustundag[a], Jeri La Neice Gill[a], Nikolas Rasmussen[a], Blake Saurenmann[a], Joe Bracerra[a], Michael Fowler[a], Hailey White[a] and Marconi Azadah[a]

[*a] Corresponding Author, Professor, # 0540, Department of Chemistry Geosciences and Physics, Tarleton State University, Stephenville, TX 76402, USA. srinivasan@tarleton.edu

[b] Department of Biological Sciences, Tarleton State University, Stephenville, TX 76402, USA.

[c] Department of Chemistry and Biochemistry, University of Texas at Dallas, TX. 75080, USA.

Key words: Microplastics, plant-derived polysaccharides, simulated water, flocculation, polymer bridging, water treatment, gums and mucilage

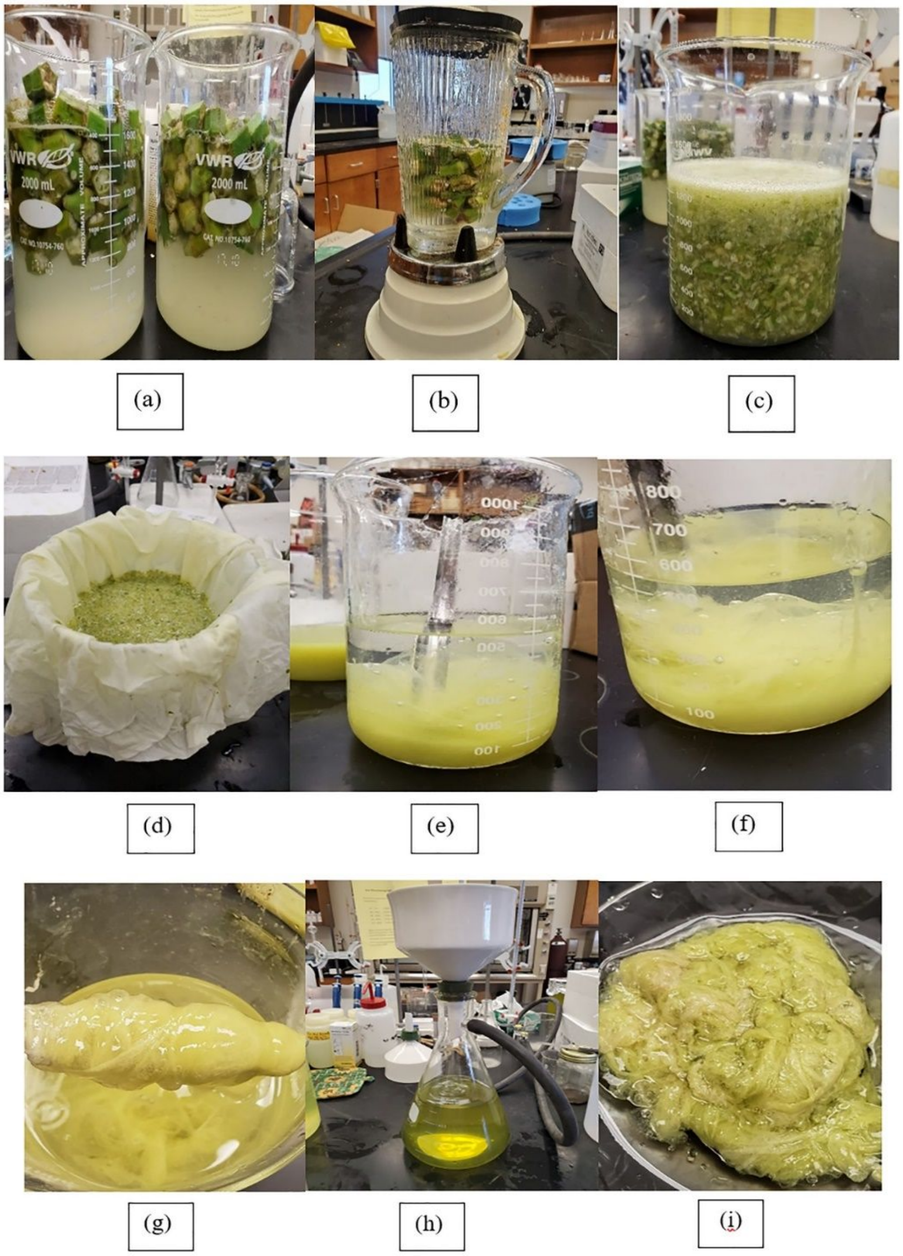

Figure S1: Plant-derived polysaccharide extrcation (a) Okra fruit cut and soaked in DI water overnight , (b) and (c) blended into fine pieces, (d) polymer extraction using muslin cloth, (e), (f), and (g) precipation and extraction of okra polymer with alcohol, (h) vacuum filtration, and (i) acetone treatment.

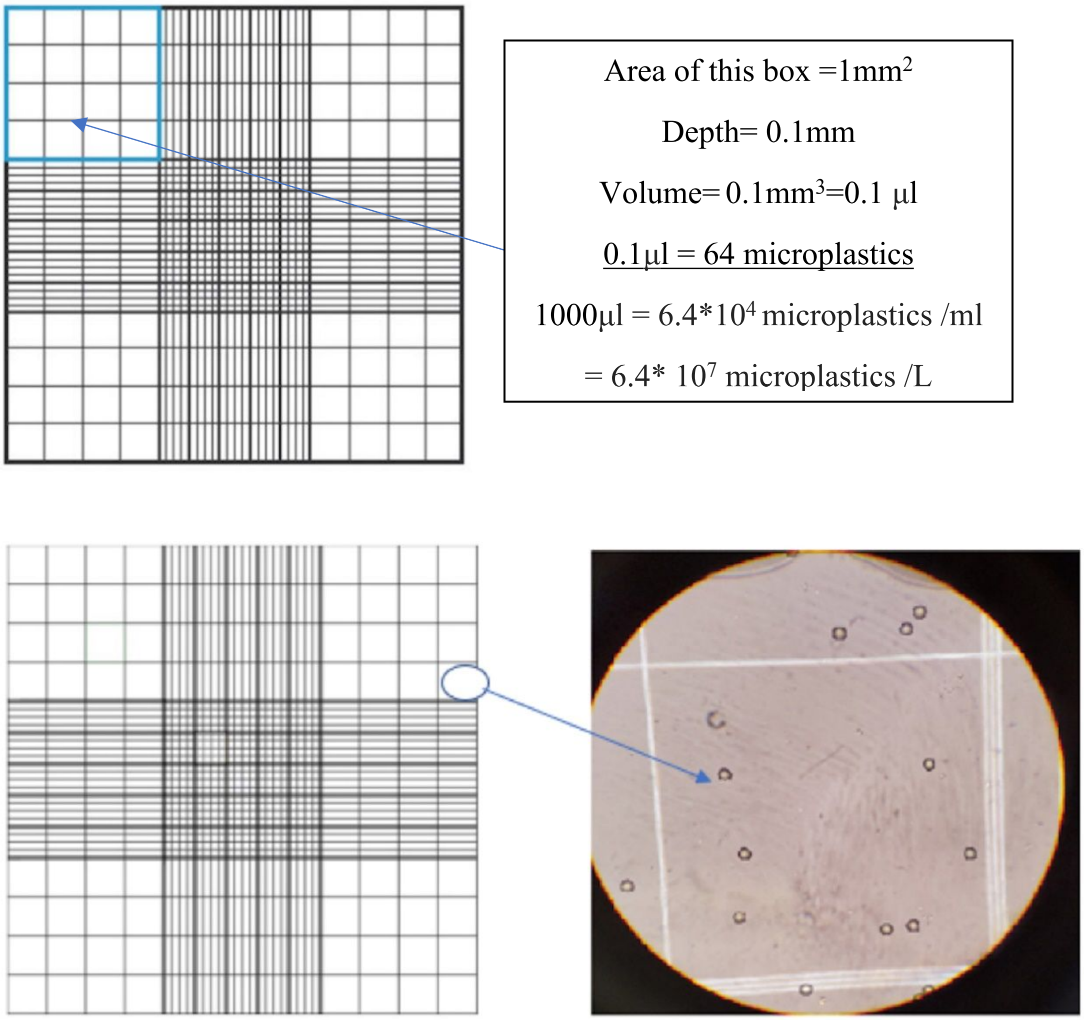

Figure S2: Total number of microplastics per millitre and microplastics count using a hemocytometer using an optical microscope at 40X magnification

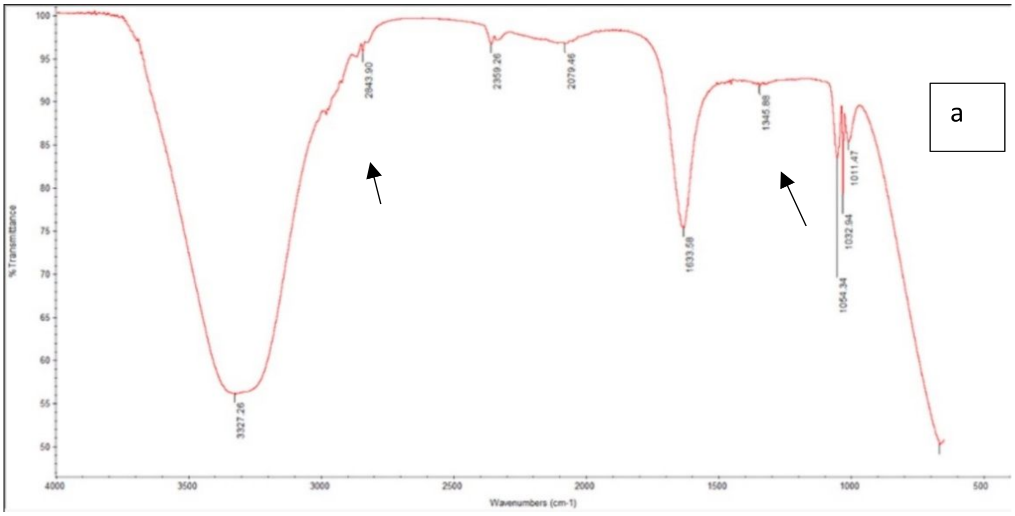
a
% Transmittance
Wavenumbers (cm-1)
3327.26
2843.90
2359.26
2079.46
1633.58
1345.86
1054.34
1032.94
1011.47

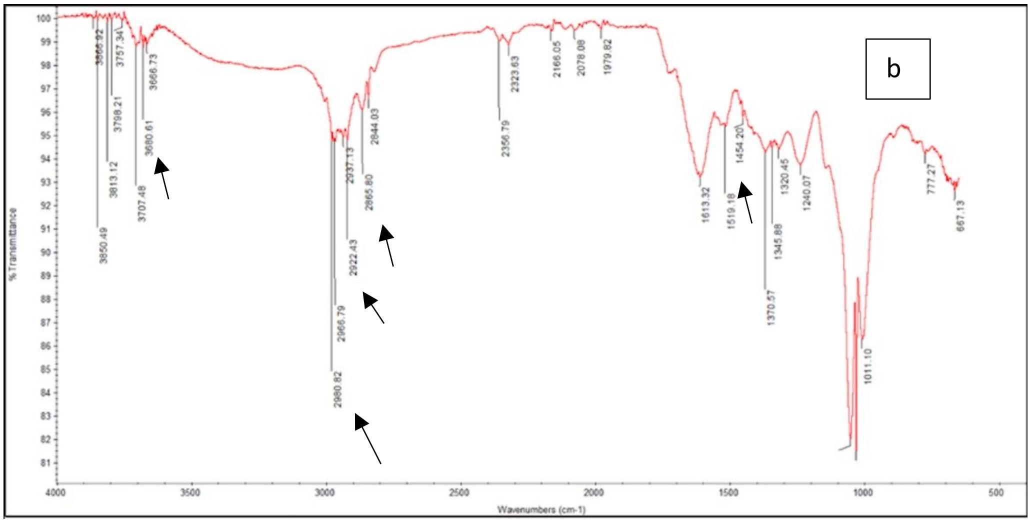
b
% Transmittance
Wavenumbers (cm-1)
3865.92
3850.49
3813.12
3798.21
3757.34
3707.48
3680.61
3666.73
2980.82
2966.79
2937.13
2922.43
2865.80
2844.03
2356.79
2323.63
2166.05
2078.08
1979.82
1613.32
1519.18
1454.20
1370.57
1345.88
1320.45
1240.07
1011.10
777.27
667.13

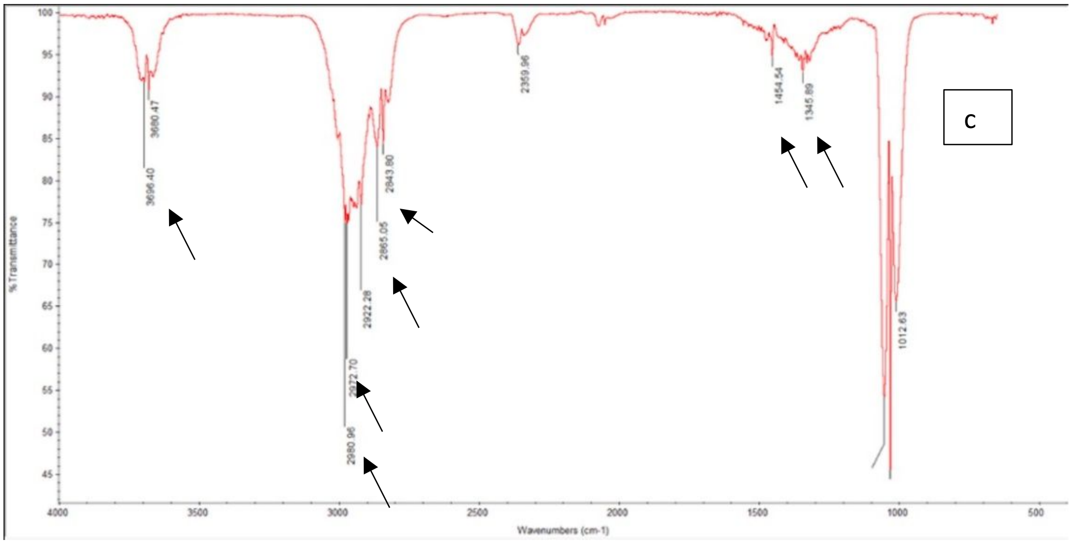
c
% Transmittance
Wavenumbers (cm-1)
3696.40
3680.47
2980.96
2972.70
2922.28
2865.05
2843.80
2359.96
1454.54
1345.89
1012.63

Figure S3: (a) FTIR spectra of okra polymer (b) FTIR spectra of polystyrene (c) FTIR spectra of okra polymer. This shows the interaction between okra polymer and microplastics at 60 min in the treated water sample.
